# Osteopontin promotes hepatocellular carcinoma progression through inducing JAK2/STAT3/NOX1-mediated ROS production

**DOI:** 10.1038/s41419-022-04806-9

**Published:** 2022-04-13

**Authors:** Qipeng Wu, Le Li, Chunmeng Miao, Muhammad Hasnat, Lixin Sun, Zhenzhou Jiang, Luyong Zhang

**Affiliations:** 1 New drug screening center, State Key Laboratory of Natural Medicines, Jiangsu Key Laboratory of Druggability of Biopharmaceuticals, China Pharmaceutical University, Nanjing, 210009 China; 2 Institute of Pharmaceutical Sciences, University of Veterinary and Animal Sciences, Lahore, 54000 Pakistan; 3 Key Laboratory of Drug Quality Control and Pharmacovigilance, Ministry of Education, China Pharmaceutical University, Nanjing, 210009 China; 4 The Center for Drug Research and Development, Guangdong Pharmaceutical University, Guangzhou, 510006 China

**Keywords:** Targeted therapies, Cell growth, Cell migration

## Abstract

Osteopontin (OPN) is a multifunctional cytokine that can impact cancer progression. Therefore, it is crucial to determine the key factors involved in the biological role of OPN for the development of treatment. Here, we investigated that OPN promoted hepatocellular carcinoma (HCC) cell proliferation and migration by increasing Reactive oxygen species (ROS) production and disclosed the underlying mechanism. Knockdown of OPN suppressed ROS production in vitro and in vivo, whereas treatment with human recombinant OPN produced the opposite effect. N-Acetyl-L-cysteine (NAC, ROS scavenger) partially blocked HCC cell proliferation and migration induced by OPN. Mechanistically, OPN induced ROS production in HCC cells by upregulating the expression of NADPH oxidase 1 (NOX1). NOX1 knockdown in HCC cells partially abrogated the cell proliferation and migration induced by OPN. Moreover, inhibition of JAK2/STAT3 phosphorylation effectively decreased the transcription of NOX1, upregulated by OPN. In addition, NOX1 overexpression increased JAK2 and STAT3 phosphorylation by increasing ROS production, creating a positive feedback loop for stimulating JAK2/STAT3 signaling induced by OPN. This study for the first time demonstrated that HCC cells utilized OPN to generate ROS for tumor progression, and disruption of OPN/NOX1 axis might be a promising therapeutic strategy for HCC.

## Introduction

Hepatocellular carcinoma (HCC) is one of the most common malignant tumors in China. Histologically, it is the most common primary liver cancer, accounting for 85–90% of all primary liver cancers. The mortality rate of liver cancer is ranked third among other malignant tumors [1]. Although remarkable progress has been made in the diagnosis, surgery, and biotherapy of HCC, the overall prognosis of patients with HCC is still not ideal due to recurrence, metastasis, chemotherapy, and radiation. It is quite essential to further explicate the biological mechanisms of HCC malignancy aiming to develop more effective therapeutic strategies.

Osteopontin (OPN), a phosphorylated glycoprotein widely expressed in many cells and tissues, has many biological functions [2]. OPN is highly expressed in tumor tissues and is present in the serum of many patients with malignant tumors (including liver cancer) [3]. In a meta-analysis, the area under ROC curve of serum OPN in diagnosis of HCC was higher than that of alpha-fetoprotein (AFP) [4]. Therefore, OPN is a promising serum biomarker of HCC. Moreover, OPN is related to tumor grade and recurrence [5]. OPN played an important role in regulating metastasis [6, 7], proliferation [8], and immune response [9, 10] of HCC, probably involved in the activation of mitogen-activated protein kinases (MAPK), NF-kB, and PI3K/Akt pathways in HCC cells. However, it is still not clear how the downstream effect of the OPN-induced signaling pathway promotes the growth and metastases of HCC.

Cancer cells have inherent elevated reactive oxygen species (ROS) level compared with normal cells [11, 12]. The moderate increase in ROS level promotes the survival and progression of cancer cells by activating various oncogenic signaling pathways [13]. Previous study has shown that OPN deficiency increased the expression of NADPH oxidase (NOX) subtypes and may induce oxidative stress in renal injury induced by angiotensin II [14]. However, pretreatment of mouse cardiomyocytes with OPN can upregulate ROS generation [15]. ROS production in myofibroblasts and vascular smooth muscle cells in OPN knockout mice was inhibited [16]. Nevertheless, the effect of OPN on ROS in HCC is not clear.

In this study, we systematically analyzed OPN-mediated changes in ROS production and assessed the role of OPN-induced ROS in cancer progression. We further revealed the mechanism of ROS production induced by OPN. In summary, our findings highlighted the role of OPN as a NOX1-induced ROS stimulator in HCC biology, which contributed to the HCC cell proliferation and migration.

## Results

### OPN-induced ROS production promotes the cell proliferation and migration of HCC cells

**Fig. 1:**
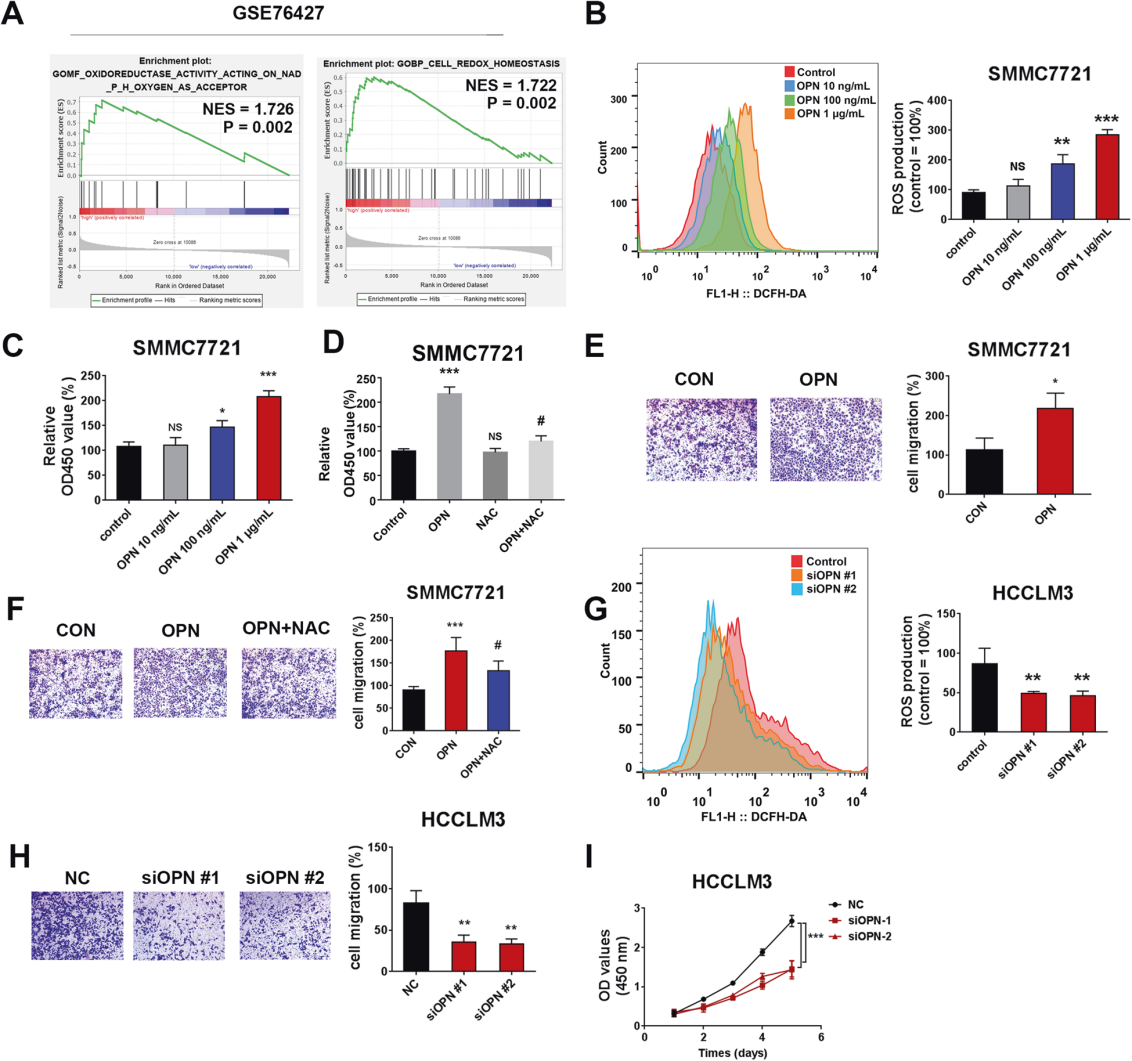
OPN-induced ROS production promotes the cell proliferation and migration of HCC cells. The results of oxidoreductase activity and cell redox homeostasis in the tumor tissues of HCC patients (data from GEO data-set GSE76427) (*n* = 115) (**A**). The level of ROS in SMMC7721 cells after treatment with different concentrations of OPN recombinant protein (10 ng/mL, 100 ng/mL, 1 μg/mL) (**B**). CCK8 and transwell assays were performed to detect the viability and migration of SMMC7721 cells (**C**, **E**). The effects on cell viability and migration were analyzed in hOPN treated SMMC7721 cells after treating them with NAC (5 mM) for 2 h (**D**, **F**). The level of ROS in HCCLM3 cells, 72 h after infecting them with siOPN (**G**). The transwell assay and growth curve were performed to detect the proliferation and migration of HCCLM3 cells (**H**, **I**). Original magnification, ×100 (scale bars: 100 μm). Data indicated mean ± SD. *n* = 3, *P < 0.05, **P < 0.01, ***P < 0.001, # Compared with hOPN treatment.

GSEA of GSE76427 showed that the high expression of OPN was correlated with oxidoreductase activity (NES = 1.726, *P* = 0.002) and cell redox homeostasis (NES = 1.722, *P* = 0.002) (Fig. 1A). To confirm whether OPN affected cellular ROS production, flow cytometric analysis of ROS-sensitive dye DCFDA was performed in HCC cells. A dose-dependent increase in DCFH-DA fluorescence after the treatment of human recombinant osteopontin (hOPN; 10 ng/mL, 100 ng/mL, 1 μg/mL) indicated an increase in ROS production in SMMC7721 cells (Fig. 1B). The results of CCK8 and transwell assays showed that hOPN increased the viability and migration of SMMC7721 cells in a dose-dependent manner (Fig. 1C, E). ROS scavenger N-acetylcysteine (NAC; 5 mM) and Butylated Hydroxyanisole (BHA; 100 μM) pretreatment effectively reduced the level of ROS in SMMC7721 cells (Fig. S4A, B). NAC (Fig. 1D, F) and BHA (Fig. S5A, B) treatment reversed the increase in viability and migration of SMMC7721 cells induced by OPN. Next, we transfected HCCLM3 cells with OPN siRNA, and western blot results revealed that OPN was significantly silenced (Fig. S3A). On the contrary, depletion of OPN effectively reduced ROS level in HCCLM3 cells (Fig. 1G). As shown in Fig. 1H, I, two OPN-siRNAs effectively inhibited the migration and proliferation of HCCLM3 cells. These results suggested that OPN induced ROS to promote the proliferation and migration of HCC cells.

### An increase in NOX1 level by OPN stimulates ROS production and promotes cell proliferation and migration in SMMC7721 and HCCLM3 cells

Mitochondria and NOXs family, including NOX1, NOX2, NOX3, NOX4, NOX5, DUOX1, and DUOX2, are two important sources of ROS production in cancer cells [17]. Therefore, we first speculated whether OPN affected cellular ROS by influencing mitochondrial ROS production. MitoSox fluorescence showed that hOPN treatment (1 μg/mL) or OPN knockdown did not show a considerable effect on the level of mitochondrial ROS in the SMMC7721 and HCCLM3 cells (Fig. S1A, C). Assessment of mitochondrial membrane potential (MMP) demonstrated that hOPN treatment or OPN deficiency did not alter the level of MMP in these cells (Fig. S1B, D).

**Fig. 2:**
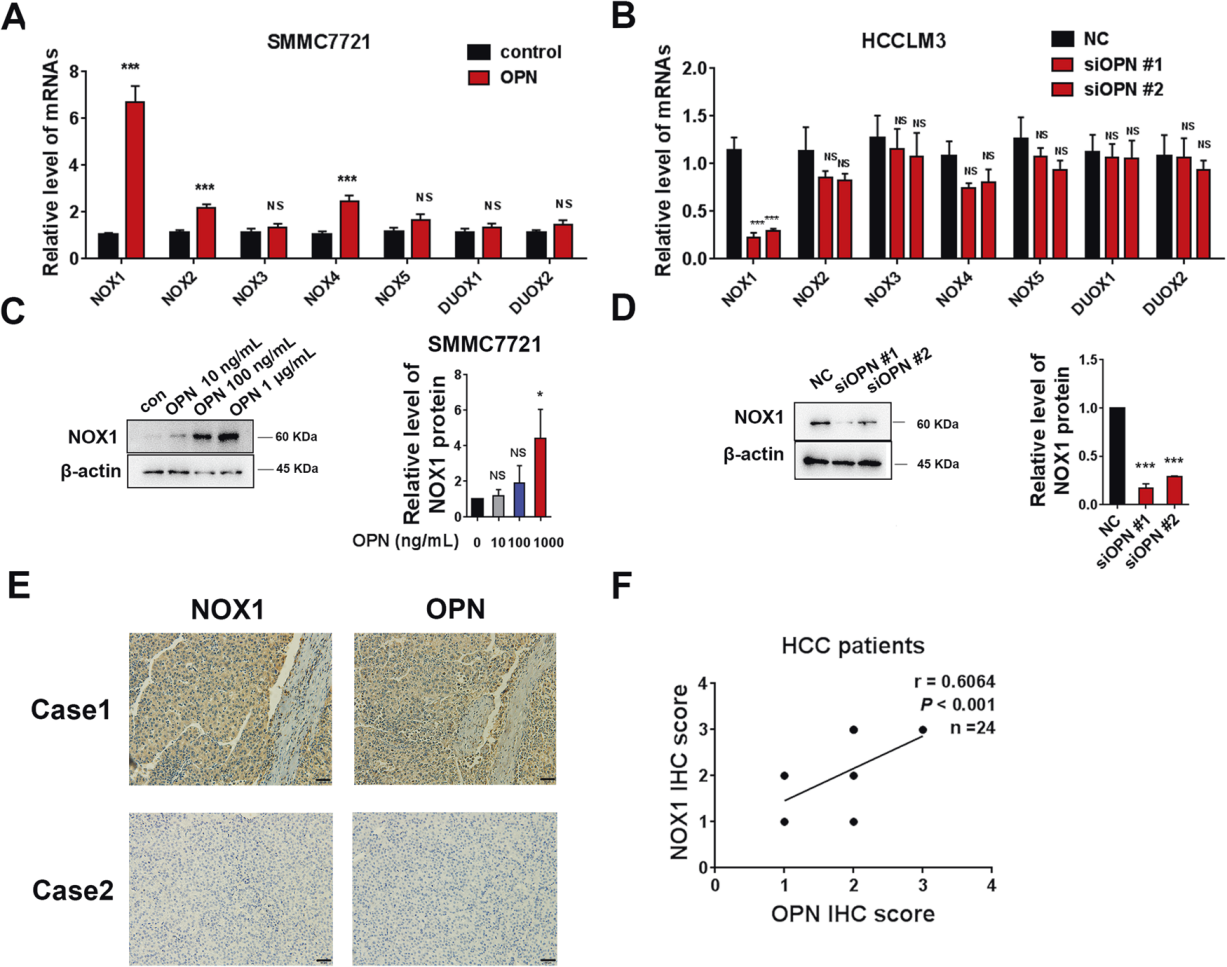
NOX1 is upregulated by OPN in HCC cells. qRT-PCR was performed to examine the effect of hOPN treatment (1 μg/mL) and OPN knockdown on the mRNA levels of NOX family (**A**, **B**). Western blot was performed to examine the effect of hOPN treatment (10 ng/mL, 100 ng/mL, 1 μg/mL) and OPN knockdown on the level of NOX1 expression (**C**, **D**). Immunohistochemical analysis of 24 human HCC samples stained with OPN and NOX1 antibodies (**E**). Original magnification, ×200 (scale bar: 50 μm). The correlation between the IHC scores of OPN and NOX1 was analyzed (**F**). Data indicated mean ± SD. *n* = 3, *P < 0.05, **P < 0.01, ***P < 0.001, NS not significant.

We further explored whether OPN-induced ROS was related to NOXs expression. As shown in Fig. 2A, B, the mRNA levels of NOX1, NOX2, and NOX4 were increased by hOPN treatment, especially NOX1. OPN knockdown decreased mRNA level of NOX1 in HCCLM3 cells. This indicated that NOX1 is the potential downstream target of OPN. Western blot analysis further confirmed that hOPN treatment led to an increase in the protein level of NOX1 in SMMC7721 cells (Fig. 2C). OPN knockdown significantly decreased the protein level of NOX1 in HCCLM3 cells (Fig. 2D). As shown in the IHC staining images of OPN and NOX1 in human HCC tissue microarray, the levels of OPN were positively correlated with levels of NOX1 in 24 HCC tumor samples (Fig. 2E). Quantification of the staining showed that these correlations were significant (*n* = 24, Pearson *r* = 0.6064, *P* < 0.001, Fig. 2F).

**Fig. 3:**
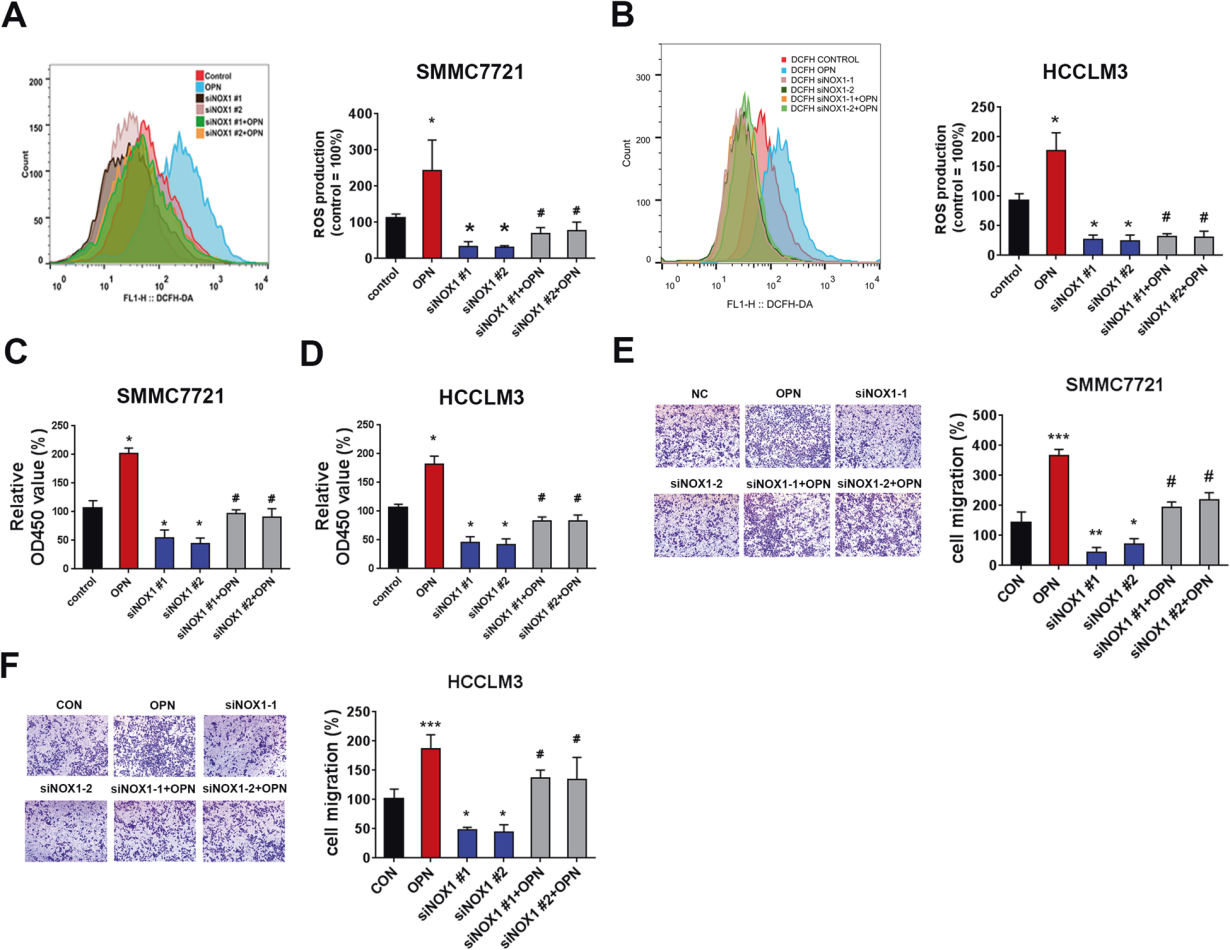
OPN stimulates ROS production and promotes cell proliferation and migration through upregulating NOX1 in HCC cells. **A,** **B** DCFH-DA fluorescence was used to evaluate total ROS production in control HCC cell, hOPN treated (1 μg/mL) HCC cells, siNOX1 HCC cells and siNOX1 HCC cells treated with hOPN. Representative images of cell viability (**C**), and cell migration (**E**) assays using SMMC7721 control, hOPN treated, siNOX1 treated, and siNOX1+OPN treated SMMC7721 cells. All the experimental conditions in (**C**, **E**) were the same as in **D**, **F**, except SMMC7721 cells were used instead of HCCLM3 cells. Original magnification, ×100. Data indicated mean ± SD. *n* = 3, *P < 0.05, **P < 0.01, ***P < 0.001, # Compared with hOPN treatment, NS not significant.

To further explore whether NOX1 mediated the function of OPN in SMMC7721 and HCCLM3 cells, the cells with NOX1 knockdown were treated with hOPN. NOX1 knockdown suppressed the ROS level that was upregulated by hOPN treatment (Fig. 3A, B). NOX1 siRNA also partially blocked the cell viability and migration promoted by hOPN (Fig. 3C–F). These results indicated that OPN regulated ROS production, cell migration, and proliferation of HCC cells through upregulating the expression of NOX1.

### JAK2/STAT3 signaling mediates OPN-induced NOX1 upregulation

**Fig. 4:**
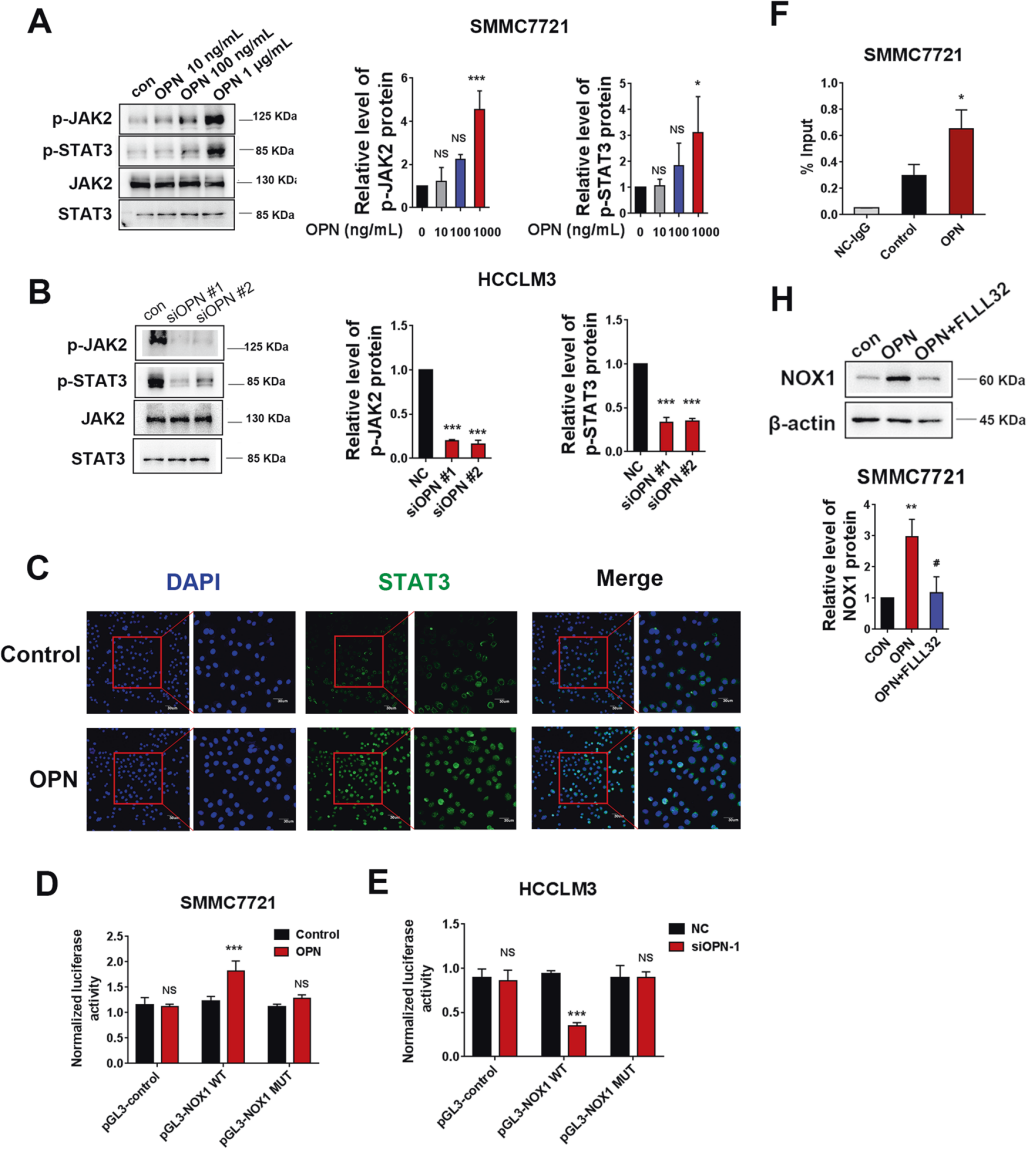
JAK2/STAT3 signaling plays a crucial role in OPN-induced NOX1 upregulation. Western blot was performed to examine the effect of hOPN treatment (10 ng/mL, 100 ng/mL, 1 μg/mL) and OPN knockdown on the levels of pJAK2 and pSTAT3 expression (**A**, **B**). SMMC7721 cells were incubated with PBS or 1 μg/mL OPN and fluorescence-stained with STAT3 antibody and DAPI (**C**). The NOX1 promoter construct pGL3-NOX1 (−200/+1) was transiently co-transfected with pRL-SV40 into SMMC7721 cells treated with OPN or HCCLM3 cells transfected with siOPN. The dual-luciferase activity was determined (**D**, **E**). CHIP assays in SMMC7721 cells incubated with hOPN or PBS. Chromatin was immunoprecipitated with anti-STAT3 or anti-IgG, and the amounts of precipitated NOX1 promoter fragments were determined by qPCR using the specific primer sets (**F**). The levels of NOX1 protein were analyzed in hOPN treated SMMC7721 cells and hOPN treated SMMC7721 cells after treating them with FLLL32 (10 μM, 8 h) (**H**). Data indicated mean ± SD. *n* = 3, *P < 0.05, **P < 0.01, ***P < 0.001, # Compared with hOPN treatment.

To further validate the effect of OPN on NOX1, HCC cells were treated with hOPN or siOPN. Western blot analysis showed that hOPN treatment led to an increase in JAK2 and STAT3 phosphorylation in SMMC7721 cells (Fig. 4A). OPN knockdown resulted in a decrease in the level of pJAK2 and pSTAT3 in HCCLM3 cells (Fig. 4B). OPN also enhanced the nuclear translocation of STAT3 (Fig. 4C). Next, we investigated the effect of STAT3 activation on the elevated expression of NOX1 in hOPN-treated cells. Dual-luciferase reporter assays revealed that hOPN significantly increased the luciferase activity of pGL3-NOX1 WT. The STAT3-binding site was then modified using site-directed mutagenesis to create M (STAT3), which reduced NOX1 promoter activity in hOPN-treated cells (Fig. 4D). On the contrary, OPN knockdown effectively reduced NOX1 promoter activity but did not affect the activity of NOX1 promoter with STAT3-binding site mutation (Fig. 4E). Consistent with this, CHIP-qPCR revealed more binding of STAT3 to the NOX1 promoter in hOPN-treated cells than in control cells (Fig. 4F). Furthermore, SMMC7721 cells receiving hOPN treatment were pretreated with a JAK2/STAT3 inhibitor FLLL32. FLLL32 completely blocked NOX1 expression that was upregulated by hOPN (Fig. 4H). These findings indicated that OPN upregulated NOX1 through JAK2/STAT3 signaling.

### NOX1-induced ROS activity promotes the proliferation and migration of HCC cells

**Fig. 5:**
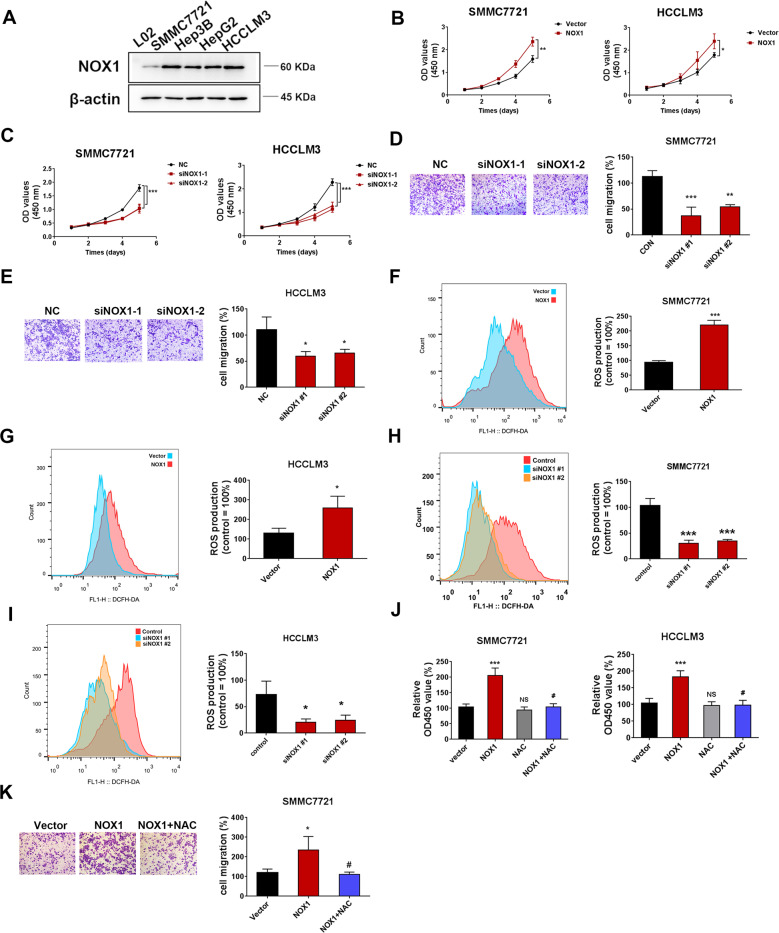
NOX1-induced ROS activity promotes the proliferation and migration of HCC cells. Western blot analysis of NOX1 expression in 4 HCC cancer cell lines (**A**). SMMC7721 and HCCLM3 cells were transfected with siNOX1 or the negative control, as well as NOX1 overexpression plasmid or vector control. **B**, **C** growth curve and **D**, **E** transwell assays were performed to detect the proliferation and migration of SMMC7721 and HCCLM3 cells. DCFH-DA fluorescence was used to evaluate the total ROS production in two cell lines mentioned above (**F**–**I**). The levels of cell viability and migration were analyzed in NOX1 overexpressed HCC cells and NOX1 overexpressed HCC cells with NAC (5 mM) pretreatment for 2 h (**J**, **K**). Original magnification, ×100 (scale bars: 100 μm). Data indicated mean ± SD. *n* = 3, *P < 0.05, **P < 0.01, ***P < 0.001, # Compared with NOX1 overexpression.

To further explore the influence of NOX1 on HCC cells, we determined the NOX1 expression phenotype in HCC cell lines (SMMC7721, Hep3B, HepG2, and HCCLM3) and normal hepatic L-02 cells. Western blot results revealed that NOX1 expression was markedly higher in HCC cell lines than that in the normal hepatocytes (Fig. 5A). We transfected HCCLM3 and SMMC7721 cells with the NOX1 overexpression plasmid and NOX1 siRNA. Western blot results showed that NOX1 overexpression plasmid and NOX1 siRNA were efficiently transfected into HCC cells (Fig. S3B–E). NOX1 overexpression increased the proliferation of HCCLM3 and SMMC7721 cells as indicated by CCK8 assay (Fig. 5B). On the contrary, NOX1 siRNA effectively inhibited the proliferation of HCCLM3 and SMMC7721 cells (Fig. 5C). Also, NOX1 siRNA effectively decreased SMMC7721 and HCCLM3 cells migration by transwell assay (Fig. 5D, E). DCFH-DA fluorescence showed that NOX1 overexpression increased ROS level (Fig. 5F, G). Subsequently, NOX1 knockdown led to a decrease in ROS level (Fig. 5H, I). Furthermore, we explored whether NOX1-induced ROS activity affected the proliferation and migration of HCC cells. The cells were transfected with NOX1 overexpression plasmid for 72 h in the presence or absence of NAC (5 mM) pretreatment. NAC treatment also effectively reduced ROS production in HCCLM3 cells (Fig. S4C). NOX1 overexpression significantly increased the cell viability of SMMC7721 and HCCLM3 cells. NAC treatment abrogated the increase in proliferation induced by NOX1 (Fig. 5J). NAC treatment also abrogated the increase in the migration of SMMC7721 cells induced by NOX1 (Fig. 5K). Taken together, these outcomes demonstrated that NOX1-induced ROS activity promoted the proliferation and migration of HCC cells.

### NOX1-induced ROS stimulates JAK2/STAT3 pathway and amplifies the effect of JAK2/STAT3 by OPN in HCC cells

**Fig. 6:**
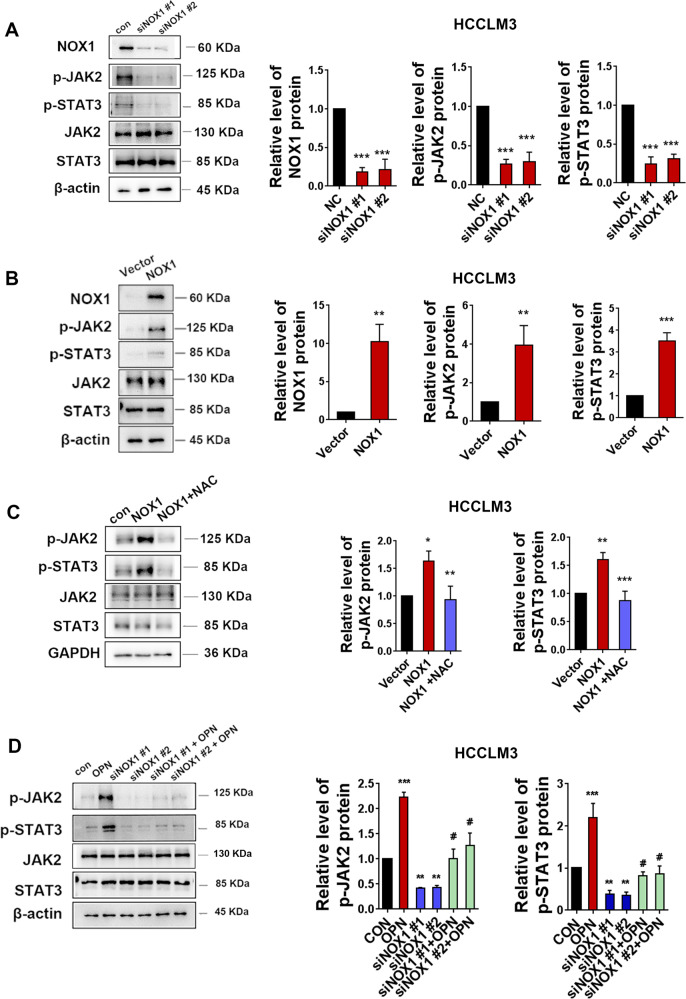
NOX1-induced ROS stimulates JAK2/STAT3 pathway and amplifies the effect of JAK2/STAT3 by OPN in HCCLM3 cells. HCCLM3 cells were transfected with siNOX1 or negative control. **A** Western blot analysis of NOX1, pJAK2, and pSTAT3 expressions was performed after transfection. The levels of NOX1, pJAK2, and pSTAT3 proteins were analyzed after transfection with NOX1 overexpression plasmid or vector control (**B**). The levels of pJAK2 and pSTAT3 proteins were analyzed in NOX1 overexpressed HCCLM3 cells and NAC treated NOX1 overexpressed HCCLM3 cells (**C**). Western blot analysis was carried out for the levels of pJAK2 and pSTAT3 proteins of control HCCLM3 cell, hOPN treated HCCLM3 cells, siNOX1 HCCLM3 cells, and siNOX1 HCCLM3 cells treated with hOPN (**D**). Data indicated mean ± SD. *n* = 3, *P < 0.05, **P < 0.01, ***P < 0.001, # Compared with NOX1 overexpression or hOPN treatment.

Next, we explored whether NOX1 upregulation by OPN could stimulate JAK2/STAT3 pathway activation. As shown in Fig. 6A and Fig. S2A, NOX1 knockdown effectively reduced NOX1 protein level and reduced JAK2 and STAT3 phosphorylation without disturbing the total JAK2 and STAT3 protein levels in HCCLM3 and SMMC7721 cells. Furthermore, NOX1 overexpression significantly enhanced JAK2 and STAT3 phosphorylation in HCCLM3 and SMMC7721 cells (Fig. 6B and Fig. S2B). NAC treatment blocked the activation of JAK2/STAT3 that was induced by NOX1 in these cells (Fig. 6C and Fig. S2C). Together, the data demonstrated that NOX1-induced ROS promoted the activation of JAK2/STAT3 signaling in HCC cells.

SMMC7721 and HCCLM3 cells were pre-transfected with NOX1 siRNA followed by hOPN treatment. As shown in Fig. 6D and Fig. S2D, hOPN treatment substantially activated the JAK2/STAT3 pathway, whereas siNOX1 could partially reverse OPN-mediated JAK2/STAT3 activation in these cells. These results showed that NOX1 increased JAK2 and STAT3 phosphorylation by producing ROS, amplifying JAK2/STAT3 signaling induced by OPN.

### OPN depletion suppresses ROS production, NOX1 expression, JAK2/STAT3 signaling, and xenograft growth in vivo

**Fig. 7:**
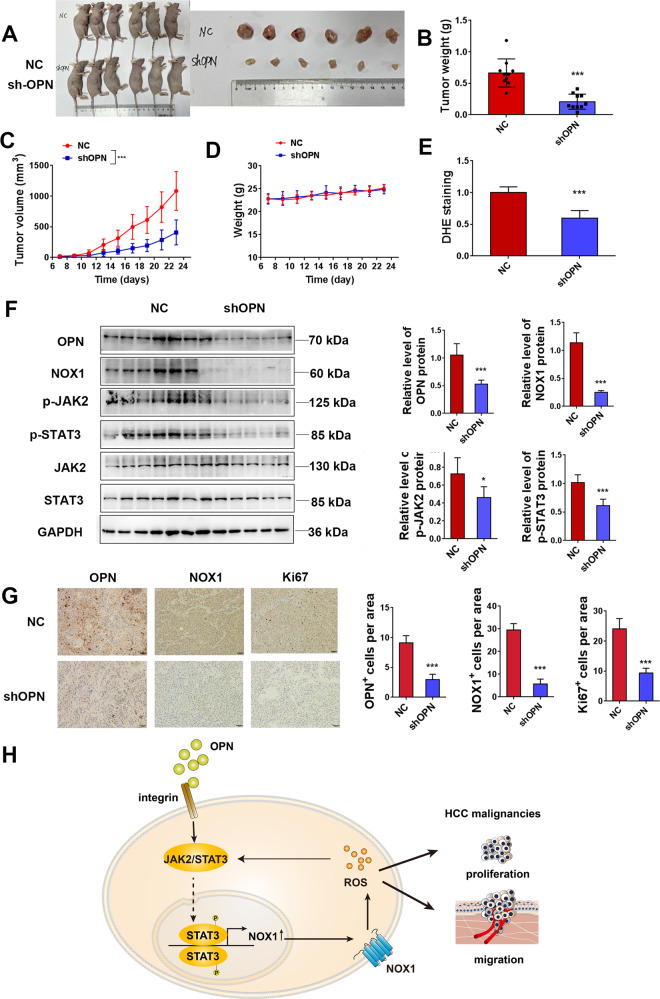
OPN depletion suppresses ROS production, NOX1 expression, and xenograft growth in vivo. **A**–**D** Nude mice (ten per group) were injected with negative control or shOPN HCCLM3 cells. The mice were killed after 23 days of injection, and the tumors were excised and weighed (**B**). The volume of the tumors (**C**) were measured every other day for an indicated 23-day period. **D** The effect of OPN knockdown on the body weight of mice. **E** The level of ROS in the tumor tissue was assayed by DHE. **F** Western blot analyses of NOX1, pJAK2, and pSTAT3 expressions in the subcutaneous tumor (*n* = 6). **G** Immunohistochemistry of tumor tissues stained with OPN, NOX1, and Ki67 antibodies (*n* = 6). Quantification is on the right side. Original magnification, ×200 (scale bars: 50 μm). Data indicated mean ± SD. *P < 0.05, **P < 0.01, ***P < 0.001. **H** Schematic representation of the role and mechanism of OPN-induced ROS in HCC.

After in vitro evaluation, we next investigated the function of OPN in HCC progression in vivo. shOPN HCCLM3 cells and negative control (NC) HCCLM3 cells were subcutaneously injected into 5-week-old nude mice. OPN knockdown significantly decreased the tumor growth rate, tumor size, and tumor weight (Fig. 7A–C). There were no significant changes in body weight of the mice, indicating that shOPN did not affect eating or drinking behaviors of tumor-bearing mice during treatment (Fig. 7D). To figure out the mechanisms responsible for the tumor suppression, we examined the influence of OPN knockdown on cell proliferation in vitro. Part of the allografts was made into cell suspension, and ROS activity was recorded. ROS activity in the shOPN group was significantly reduced by DHE assay compared with the NC group (Fig. 7E). shOPN significantly reduced the protein level of NOX1 and phosphorylation of JAK2/STAT3 (Fig. 7F). IHC staining showed that shOPN group had lower expression of NOX1 and Ki67 compared with the NC group (Fig. 7G). All of these findings together suggested that OPN promoted cancer growth, ROS production, NOX1 expression, and phosphorylation of JAK2/STAT3 in vivo.

## Discussion

In this study, we observed that OPN could stimulate ROS level in HCC cells. OPN promoted the proliferation and migration of HCC cells by increasing ROS. We further demonstrated the mechanism of ROS production induced by OPN. OPN-induced ROS generation was enhanced by NOX1 and we further proved that OPN increased NOX1 expression through JAK2/STAT3 pathway. Confirmatory experiments on human HCC tissues showed that OPN expression had a certain correlation with NOX1 expression. Together, our findings provide a new insight into the functions of OPN in HCC and elucidate its mechanism. OPN/NOX1 axis may be a potential therapeutic target for HCC.

ROS are well known to play crucial roles in various biological responses. Their role either promotes or limits cancer cell proliferation and migration under different conditions. Appropriate and excessive ROS levels can lead to contrasting consequences [18, 19]. Previous studies reported that OPN is crucial for the regulation of ROS in renal injury induced by angiotensin II and in aortic mesenchymal cells [14, 16, 20]. However, whether OPN upregulated the ROS generation in HCC remained unknown. Here for the first time, we demonstrate that OPN acts as the upstream regulator of redox signaling in HCC cells. Furthermore, scavenging ROS using antioxidants suppresses OPN-induced oxidative stress and tumor progression.

ROS is mainly produced by NOX family members (NOX1–5, DUOX1, and DUOX2) [21]. In this study, we found that the expression of NOX1 mRNA in HCC cells with high OPN expression was higher than that in HCC cells with low OPN expression. NOX1 knockdown decreased ROS level in hOPN treated HCC cells. STAT3 binds to the NOX1 promoter and induces NOX1 promoter activity [22]. The dual-luciferase assay showed that the binding of STAT3 to NOX1 promoter was higher in cells with high OPN expression than that in cells with low OPN expression. Interestingly, we found that the activity of STAT3 phosphorylation in the cells with high OPN expression was higher than that in the cells with low OPN expression. In conclusion, high expression of NOX1 induced by STAT3 activation is responsible for the high level of ROS in hOPN treated HCC cells. OPN is an important regulator of HCC cell proliferation, migration, and invasion [23]. Our findings provide a new mechanism for OPN in the regulation of ROS, involving upregulation of NOX1.

It has been reported that NOX1 is an abnormal expression of NOX subunit in tumor cells, which plays a crucial role in cell proliferation, apoptosis, and cell migration of colon cancer [24], gastric cancer [25], skin cancer [26] and melanoma [27]. Besides, NOX1 has also been found to be highly expressed in HCC, which is consistent with previous studies [28, 29]. However, we found that the different OPN expressing cells had similar NOX1 protein levels. NOX1 is induced by various cellular effectors, which increase the expression of NOX1 mRNA and protein, thereby stimulating the O^2•−^ production [30]. Moreover, the gene expression profiles are different across different cell lines. Therefore, the expression of NOX1 may be regulated not only by OPN but also by other proteins. Our data showed that NOX1 knockdown could decrease cell viability and cell migration. These outcomes provide evidence that NOX1 is essential for the tumorigenic phenotype of HCC. A previous study has shown that NOX1 promotes cell proliferation through ROS production [31]. We found that treatment of HCC cells with ROS scavenger showed a decrease in cell growth and migration. These results suggested that NOX1 regulates cancer progression through ROS production in HCC. Besides, ROS generation by NOXs activated JAK2/STAT3 pathway [32]. Many studies have reported that the activation of JAK2/STAT3 pathway contributes to the progression of various cancers [33]. Furthermore, we found that NOX1-induced ROS could promote the phosphorylation of STAT3 in HCC cells. OPN-induced STAT3 phosphorylation was partially suppressed by NOX1 knockdown. Therefore, all the findings together revealed that OPN-induced NOX1 created a positive feedback loop with stimulation of JAK2/STAT3 signaling by OPN in HCC cells.

Moderate level of ROS can indeed maintain the malignant progression of tumors, while persistent or sharply elevated ROS production induces tumor cell apoptosis. However, in general, cancer molecules or cancer signaling pathways-induced moderate level of ROS effectively maintains the survival of tumor cells, and cannot promote the apoptosis of tumor cells. In case of strong exogenous stimulation, such as chemotherapeutic drugs or targeted drugs, toxic level of ROS will be overproduced. This violent and rapid upregulation of ROS induces tumor cell death. Observing the relationship between upper/lower threshold of ROS and cell survival/death of tumor cells is a topic of constant discussion in tumor biology [34, 35]. In this study, we believe that the generation of ROS by the OPN/NOX1 axis can maintain tumor cell survival and malignant progression. Our supplementary experiments (Fig. S6) also confirmed that regulating this signaling pathway did not induce apoptosis.

In summary, we confirm that OPN can increase the ROS level of HCC cells through JAK2/ STAT3/NOX1 signaling, and promotes HCC progression through ROS. Above all, our study shows a mechanistic link between OPN and ROS and establishes OPN/NOX1 axis as attractive therapeutic targets for HCC. Specifically, our data also suggest that strategies to block ROS signaling via NOX1 inhibition in patients with OPN^high^ HCC may result in better efficacy compared with patients having OPN^low^.

## Materials and methods

### Antibodies and reagents

Rabbit polyclonal anti-Ki67 (1:1000, 9027), anti-phospho-JAK2 (Tyr-1007/1008, 1:1000, 3776), anti-phospho-STAT3 (Tyr-705, 1:1000, 9145) were obtained from Cell Signaling Technology (MA, USA). Rabbit polyclonal anti-JAK2 (1:1000, A19629), and anti-STAT3 (1:1000, A11185) were purchased from Abclonal (Wuhan, China). Anti-NOX1 (1:1000, GTX103888) was collected from Genetex (CA, USA). Rabbit polyclonal anti-OPN (1:1000, ab214050) was obtained from Abcam (Cambridge, UK). The human recombinant OPN, FLLL32, NAC, and BHA were purchased from Sinobiological (Beijing, China), MCE (Shanghai, China), and Sigma-Aldrich (St Louis, USA), respectively.

### Cell culture and transfection

The human hepatocellular carcinoma cell lines SMMC7721 (weak OPN expression [36]) and HCCLM3 (high OPN expression [37]) were obtained from the National Collection of Authenticated Cell Cultures (Shanghai, China). These cell lines were cultured in RPMI medium1640 and DMEM respectively, and supplemented with 10% fetal bovine serum in a humidified atmosphere at 37 °C, having 5% CO_2_.

To knock down OPN and NOX1 by siRNA, cells were cultured at 50%-60% confluence and transfected for 72 h with OPN, NOX1 siRNA, or negative control (NC). All transfections were performed using lipofectamine 3000 (Invitrogen, USA) according to the manufacturer’s instructions. siRNA sequences are as follows: siOPN-1: 5'GGACAGTTATGAAACGAGT'3, siOPN-2: 5'CGAGGAGTTGAATGGTGCATA'3; siNOX1–1: 5'CAAGCTGGTGGCCTATATGAT'3, siNOX1–2: 5'GAGATGTGGGATGATCGTGAC'3. shRNA or scrambled control-shRNA were constructed using a lentiviral shRNA technique. The target sequence was as follows: sh-NC: CCTAAGGTTAAGTCGCCCTCG shOPN: 5'GGCTGATTCTGGAAGTTCTGA'3. NOX1 overexpression was generated with pcDNA3.1-NOX1 plasmids (accession no.NM_007052.5).

### Gene set enrichment analysis (GSEA)

Gene expression profiles, analyzed in this study, were downloaded from GENE EXPRESSION OMNIBUS (GEO, http://www.ncbi.nlm.nih.gov/geo). Data from GEO series GSE76427 and GSE133608 were analyzed using Gene Set Enrichment Analysis (GSEA). GSEA v4.0.2 software was used to identify functional associations of the expression profiles.

### Measurement of intracellular and mitochondrial ROS levels

Intracellular reactive oxygen species (ROS) and mitochondrial ROS were measured by dichloro-dihydro-fluorescein diacetate (DCFH-DA) and MitoSOX™ Red assay (YEASEN, Shanghai, China), respectively. Briefly, cells were incubated in serum-free medium with DCFH-DA (10 μM) for 30 min or MitoSOX™ Red (5 μM) for 15 min at 37 °C. Relative fluorescence intensity was recorded using a flow cytometer and microplate reader.

### Cell proliferation/viability assays

For cell proliferation assay, an equal number of the cells were dispersed in 96-well plates after carrying out transfections at 24 h and allowed to grow for 5 days in order to determine a growth curve. Cell viability was measured every 24 h by using CCK8, in a multi-detection microplate reader (Thermo, MA, USA) at 450 nm. These experiments were repeated three times independently.

Cells/transfected cells seeded in 96-well white plates were incubated with human recombinant osteopontin for 4 days. Cell viability was determined using the CCK8 assay. These experiments were repeated three times independently.

### Cell migration assay

Cell migration was measured using the transwell cell migration assay. For transwell migration assay, cells suspended in 200 µL serum-free DMEM or RMPI 1640 media were seeded into the upper chamber of the transwell. The number of cells was counted under a light microscope in five random fields (Olympus Corporation, Tokyo, Japan).

### Western blot analysis and qRT-PCR assay

Proteins from mice tumors and cell lysates were collected and separated on SDS-PAGE. After transferring to PVDF membrane, blocking step was performed using blocking agents. Incubation with respective primary and secondary antibodies was completed prior to protein band analysis using Gel imaging System (TANON, Beijing, China). Quantification of band intensity was carried out using ImageJ software (NIH, Bethesda, MD, USA). Total RNA was isolated by Trizol reagent (Vazyme biotech, Nanjing, China), and reverse transcription quantitative PCR was performed using hiscript qRT supermix (qPCR; Vazyme biotech). The mRNA levels were measured with the SYBR Green master mix (Vazyme Biotech). 18 s was used as an internal control. The relative quantification of each mRNA was carried out using the comparative Ct method. The sequences of primers used for PCR amplification are shown in Supplementary table. 1.

### Mitochondrial membrane potential assay

Mitochondrial membrane potential was detected using the JC1 assay kit (Beyotime, Beijing, China). The JC-1 working solution was prepared according to the manufacturer’s instructions. The cells were incubated under 5% CO_2_ at 37 °C for 20 min in the dark and washed with JC-1 buffer solution. Relative fluorescence intensity was recorded using a microplate reader.

### Luciferase reporter assay

HCC cells were co-transduced with the NOX1 reporter construct pGL3-NOX1 (−200/+1), pGL3-NOX1 mutant STAT3-binding sites (M, STAT3) [22, 38], and OPN siRNA or NOX1 reporter. Luciferase activity was measured by a dual-luciferase reporter gene assay system (YEASEN, shanghai, China) according to the manufacturer’s protocol. Results were presented as firefly luciferase activity normalized to Renilla luciferase activity.

### Immunofluorescence (IF)

Protein localization was investigated by IF. SMMC7721 cells were cultured on cover glasses in 24-well plates. The next day, the cells were treated with OPN (1 µg/mL). Next, the cells were fixed with paraformaldehyde for 15 min. The fixed cells were permeated with 0.5% Triton X-100(PBS preparation) at room temperature for 20 min. The cells were blocked with 5% goat serum albumin in 3% BSA for 1 h. STAT3 antibody (1:50) was added and incubated overnight at 4 °C. After washing with PBS, the cells were incubated with a fluorescein (FITC)-conjugated secondary antibody (1:500) for 1 h. The cells were washed with PBS, and cell nuclei were stained with DAPI. Cell images were captured by a laser scanning confocal microscope FV1000 (Olympus Corporation, Tokyo, Japan).

### Chromatin immunoprecipitation assays

Cells were washed twice with PBS and cross-linked with 1% formaldehyde at room temperature for 10 min. Cells were washed sequentially with 10 ml of ice-cold PBS. Cells were then resuspended in 0.3 ml of RIPA lysis buffer (1% SDS, 10 mM EDTA, 50 mM Tris-HCl, pH 8.1, 1× protease inhibitor cocktail) and sonicated six times for 10 s each at the 40% setting, followed by centrifugation for 10 min. Supernatants were collected and diluted in buffer (1% Triton X-100, 2 mM EDTA, 150 mM NaCl, 20 mM Tris-HCl, pH 8.1) followed by immunoclearing with 75 ng/μL of sheared salmon sperm DNA and 0.1 μg/μl of BSA and Protein A/G Magnetic Beads for 0.5 h at room temperature. Immunoprecipitation was performed for overnight at 4 °C with specific antibodies. Precipitates were washed sequentially for 10 min each in TSE I (0.1% SDS, 1% Triton X-100, 2 mM EDTA, 20 mM Tris-HCl, pH 8.1, 150 mM NaCl), TSE II (0.1% SDS, 1% Triton X-100, 2 mM EDTA, 20 mM Tris-HCl, pH 8.1, 500 mM NaCl), and buffer III (0.25 M LiCl, 1% NP-40, 1% deoxycholate, 1 mM EDTA, 10 mM Tris-HCl, pH 8.1). Precipitates were then washed three times with TE buffer and extracted three times with 1% SDS, 0.1 M NaHCO3. Eluates were pooled and heated at 65 °C for overnight to reverse the formaldehyde cross-linking. DNA fragments were purified with a DNA Extraction Mini Kit (Vazyme, Nanjing, China). Finally, the purified DNA was analyzed by qPCR. Primers for qPCR are listed in Supplementary Table. 1.

### Xenograft assay

Animal experiments were approved by the Animal Care Committee of China Pharmaceutical University (Approval No.2110748). Five-week-old female mice (BABL/c nude) were obtained from the Hangzhou Ziyuan Experimental Animal Technology Co., Ltd. BALB/c nude mice were randomly divided into two groups with ten mice per group. HCCLM3 cells (5 × 10^6^ cells in 100 μL PBS) were subcutaneously injected into the right flank of mice. Tumor growth and mice weight were monitored every other day, and were calculated according to the following formula: tumor volume = (length × width^2^)/2. At the end of the experiment, tumors were removed, photographed, and processed for immunohistochemical and western blot analyses.

### Tissue microarray (TMA) and immunohistochemistry (IHC)

TMAs containing HCC samples were purchased from Shanghai Outdo Bioth CO., Ltd (Shanghai, China). The TMA specimens were used for IHC analysis. IHC staining of TMAs was quantified using the IHC Profiler ImageJ Plugin [39], an automated digital program that quantitates the intensity of antibody staining in tissue sections. The spectral deconvolution method of DAB/hematoxylin was deployed, so that the DAB stained images could be separated from hematoxylin stained images. Then the program selects the areas in cytoplasm and/or nucleus where the marker protein is significantly expressed the most. Therefore the deconvoluted image undergoes a pixel-by-pixel analysis providing full profile along with a scoring decision. The staining intensity was scored as 1 to 4. Scoring was as follows: 1, negative; 2, low positive; 3, positive; 4, high positive.

Immunohistochemistry (IHC) IHC was performed as previously described. In brief, tumor tissues were fixed with 4% paraformaldehyde, blocked with 10% FBS, and permeabilized with Triton X-100. Sections were incubated with OPN antibody (Abcam, 1:50), NOX1 antibody (Genetex, 1:50), and Ki67 antibody (CST, 1:100) at 4 °C overnight. After washing with PBS, sections were incubated with the secondary antibodies for 1 h at room temperature. After counterstaining with hematoxylin, sections were visualized under a microscope (BX53, Olympus, Tokyo, Japan). The positive–stained area was determined using ImageJ software.

### DHE assay

To assess oxidative stress, we performed DHE (dihydroxyethidium) assay following standard procedures. Tumor tissues were treated with trypsin for 20 min to generate a single-cell suspension. Cells were incubated in serum-free medium with DHE (20 μM) for 30 min at 37 °C. Relative fluorescence intensity was recorded using microplate reader.

### Statistical analysis

All the data were expressed as mean ± standard deviation. All in vitro experiments were performed at least three times. Statistical comparison between the two groups was performed using a student *t*-test. For multiple groups, the one-way ANOVA or two-way analysis of variance followed by Dunnett’s multiple comparisons test or Tukey’s multiple comparison post-test were applied. Data were analyzed using Graphpad Prism 6.02 software (GraphPad Software, Inc., San Diego, CA). *P* < 0.05 was considered statistically significant.

## Supplementary information

Supplemental Material

Reproducibility checklist

Supplemental Material_original western blots

## Data Availability

All data needed to evaluate the conclusions in the paper are present in the paper. Additional data related to this paper may be requested from the corresponding author.
