# Supplementary material for: Osteopontin promotes hepatocellular carcinoma progression through inducing JAK2/STAT3/NOX1-mediated ROS production
Source: Cell Death Dis. 2022 Apr 13;13(4):341. doi: 10.1038/s41419-022-04806-9 (PMC9008047; doi:10.1038/s41419-022-04806-9)
Supplement: Supplementary file 1 — Supplemental Material [file 41419_2022_4806_MOESM1_ESM.docx]

**Supplementary Figures and Figure legends**

**
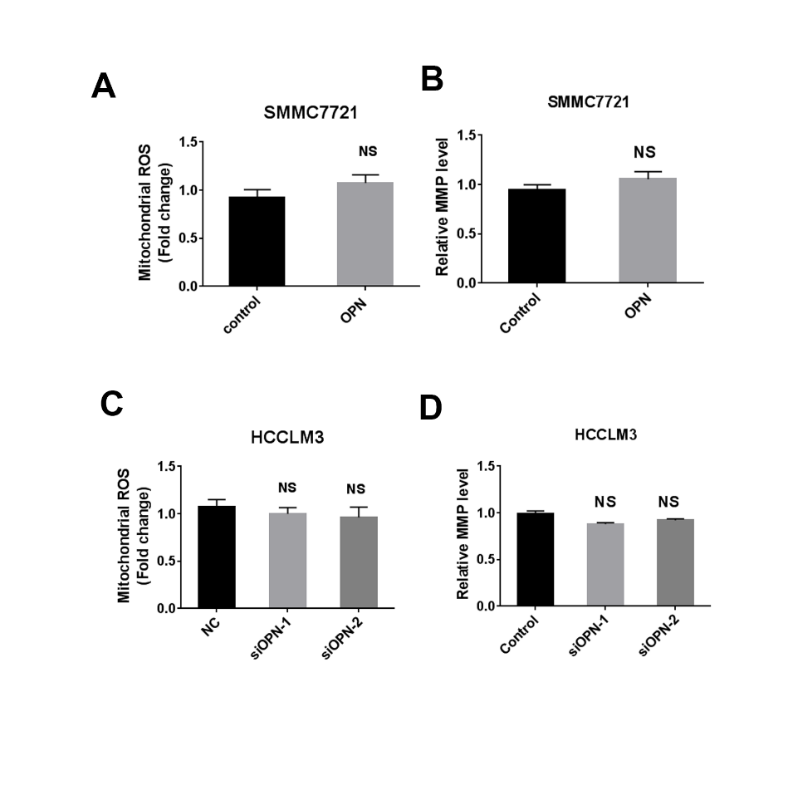
**

**Supplementary Figure 1. OPN does not affect mitochondrial ROS generation and mitochondria membrane potential (MMP)**

MitoSox was performed to examine the effect of hOPN treatment and OPN knockdown on the mitochondrial ROS level (A and C). JC-1 was performed to examine the effect of hOPN treatment and OPN knockdown on the MMP (B and D). Data indicated mean ± SD. n = 3, NS not significant.


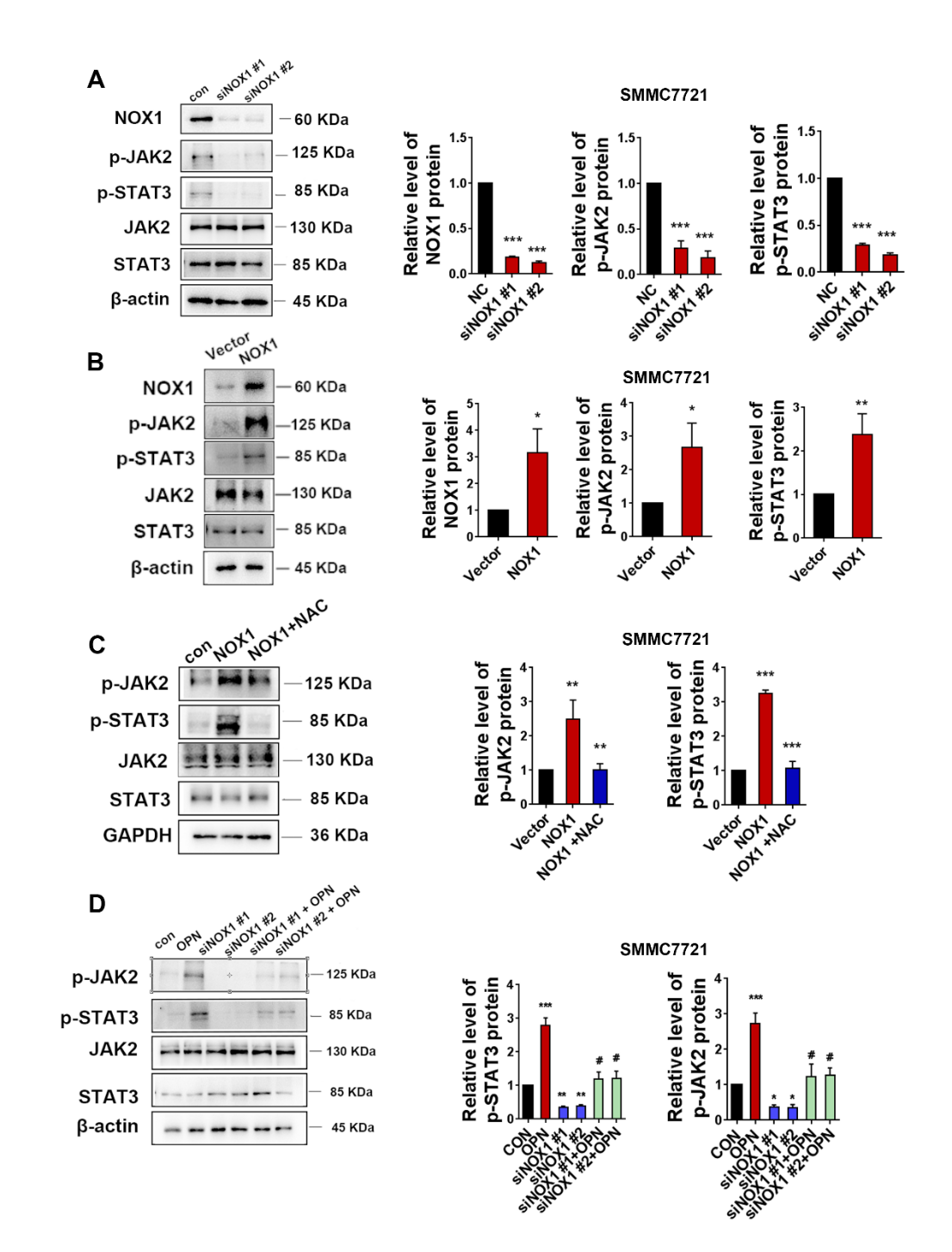


**Supplementary Figure 2. NOX1-induced ROS stimulates JAK2/STAT3 pathway and amplified the effect of JAK2/STAT3 by OPN in SMMC7721 cells**

SMMC7721 cells were infected with siNOX1 or negative control. (A) Western blot analysis of NOX1, pJAK2 and pSTAT3 expression was performed after transfection. The levels of NOX1, pJAK2 and pSTAT3 proteins were analyzed after transfecting with NOX1 overexpression plasmid or vector control (B). The levels of pJAK2 and pSTAT3 proteins were analyzed in NOX1 overexpressed SMMC7721 cells and NAC-treated NOX1 overexpressed SMMC7721 cells (C). Western blot analysis for the levels of pJAK2 and pSTAT3 proteins of control SMMC7721 cell, hOPN treated SMMC7721 cells, siNOX1 SMMC7721 cells and siNOX1 SMMC7721 cells treated with hOPN. Data indicated mean ± SD. n = 3, **P* < 0.05, ***P* < 0.01, ****P* < 0.001, # Compared with NOX1 overexpression or hOPN treatment

**
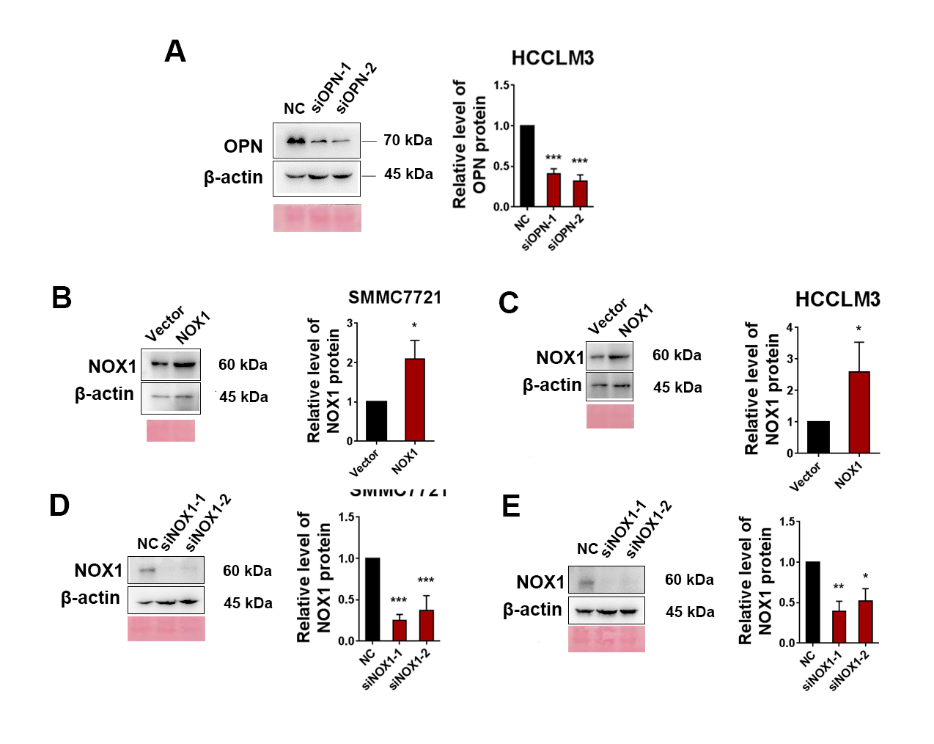
**

**Supplementary Figure 3. Western blot results for siOPN and NOX1 overexpression and siNOX1 in transfected SMMC7721 cells and HCCLM3 cells**

HCCLM3 cells were infected with siOPN or negative control. Western blot analysis of OPN expression was performed after transfection (A). The levels of NOX1 proteins were analyzed after transfecting with NOX1 overexpression plasmid or vector control (B and C). SMMC7721 and HCCLM3 cells were infected with siNOX1 or negative control. Western blot analysis of NOX1 expression was performed after transfection (D and E).


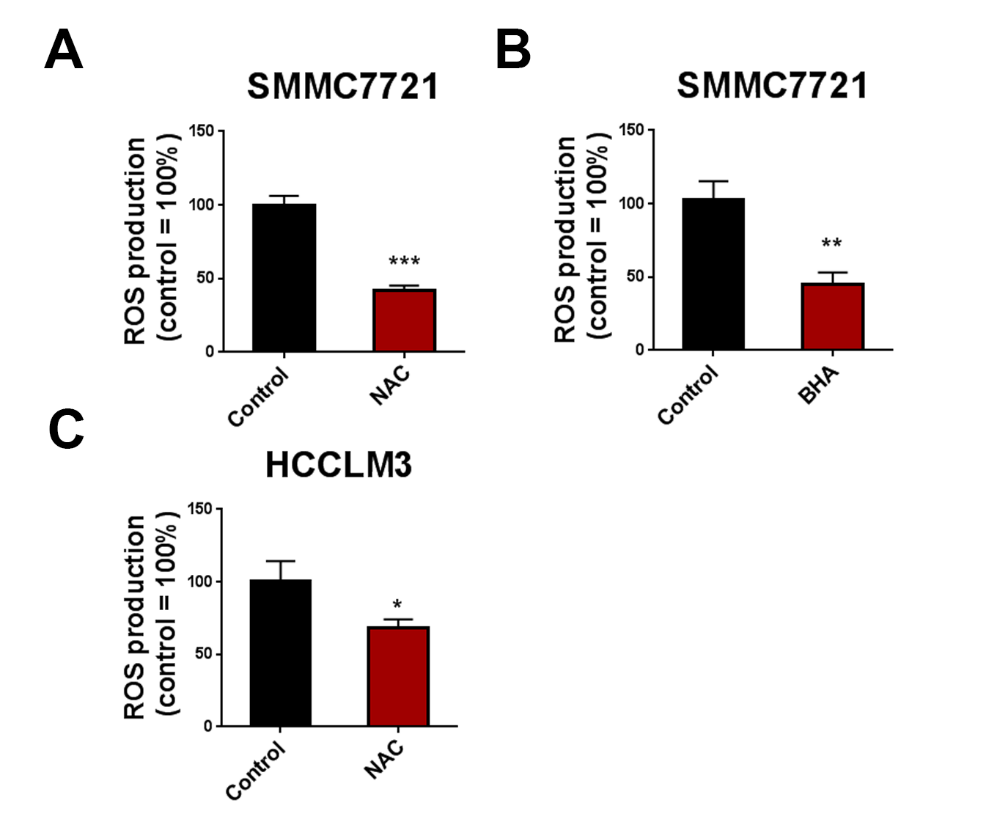


**Supplementary Figure 4. DCFH-DA results for NAC and BHA in SMMC7721 cells and HCCLM3 cells**

DCFH-DA fluorescence was used to evaluate total ROS production in SMMC7721 and HCCLM3 cells after treating with NAC (5 mM) (A and C). DCFH-DA fluorescence was used to evaluate total ROS production in SMMC7721 cells after treating with BHA (100 μM) (B). Data indicated mean ± SD. n = 3, *P < 0.05, **P < 0.01, ***P < 0.001.


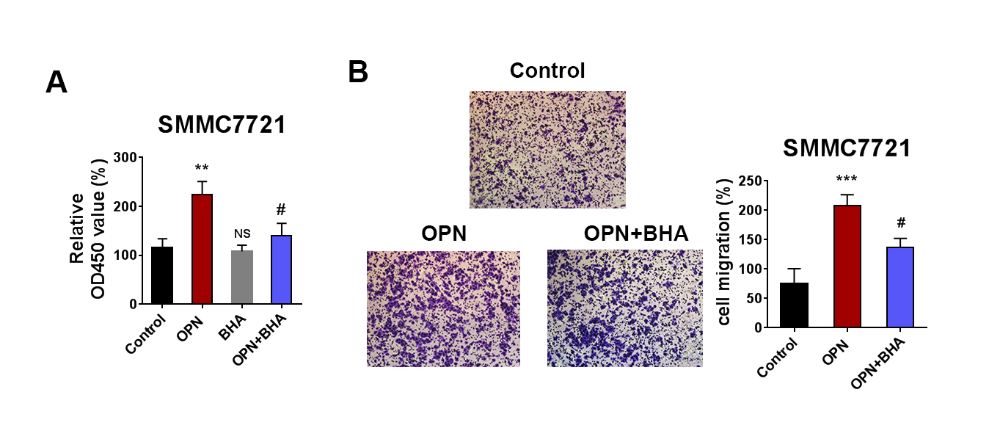


**Supplementary Figure 5. BHA blocked the increase in viability and migration of SMMC7721 cells induced by OPN**

The effects on cell viability and migration were analyzed in hOPN treated SMMC7721 cells after treating them with BHA (100 μM) for 1 h (A and B).


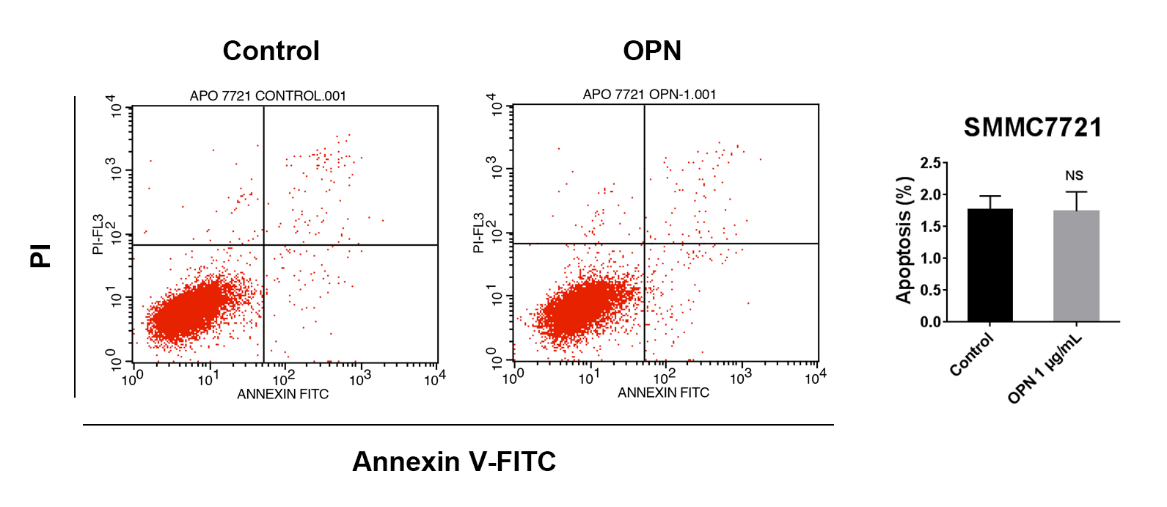


**Supplementary Figure 6. OPN did not induce apoptosis of SMMC7721 cells**

Apoptosis assays were used to detect apoptosis levels of hOPN-treated SMMC7721 and SMMC7721 cells.

**Supplementary Table. 1. The sequences of primers**

| Primer ID | Sequences |
| --- | --- |
| NOX1-F | 5’-GTCTGCTCTCTGCTTGAAT-3’ |
| NOX1-R | 5’-ATGAGATAGGCTGGAGAG-3’ |
| NOX2-F | 5’-CAAGATGCGTGGAAACTACCTAAGAT-3’ |
| NOX2-R | 5’- TCCCTGCTCCCACTAACATCA-3’ |
| NOX3-F | 5’- CCAGGGCAGTACATCTTGGT-3’ |
| NOX3-R | 5’-CCGTGTTTCCAGGGAGAGTA-3’ |
| NOX4-F | 5’-TGGCTGCCCATCTGGTGAATG-3’ |
| NOX4-R | 5’-CAGCAGCCCTCCTGAAACATGC-3’ |
| NOX5-F | 5’-TGGCTGCCCATCTGGTGAATG |
| NOX5-R | 5’-CAGCAGCCCTCCTGAAACATGC-3’ |
| DUOX1-F | 5’-TCTCTGGCTGACAAGGATGGCA-3’ |
| DUOX1-F | 5’-AGGCGAGACTTTTCCTCAGGAG-3’ |
| DUOX2-F | 5’-CAATGGCTACCTGTCCTTCCGA-3’ |
| DUOX2-R | 5’-GTCCTTGGAGAGGAAGCCATTC-3’ |
| 18S-F | 5’-CTTTGGTCGCTCGCTCCTC-3’ |
| 18S-R | 5’-CTGACCGGGTTGGTTTTGAT-3’ |
| NOX1-P-F | 5’-GGCAATGCTTCACATTAGGTCA-3’ |
| NOX1-P-R | 5’-CAAGGGTTTTACCTGTGGGGA-3’ |
