# Supplementary material for: Osteopontin promotes hepatocellular carcinoma progression through inducing JAK2/STAT3/NOX1-mediated ROS production
Source: Cell Death Dis. 2022 Apr 13;13(4):341. doi: 10.1038/s41419-022-04806-9 (PMC9008047; doi:10.1038/s41419-022-04806-9)
Supplement: Supplementary file 3 — Supplemental Material_original western blots [file 41419_2022_4806_MOESM3_ESM.docx]

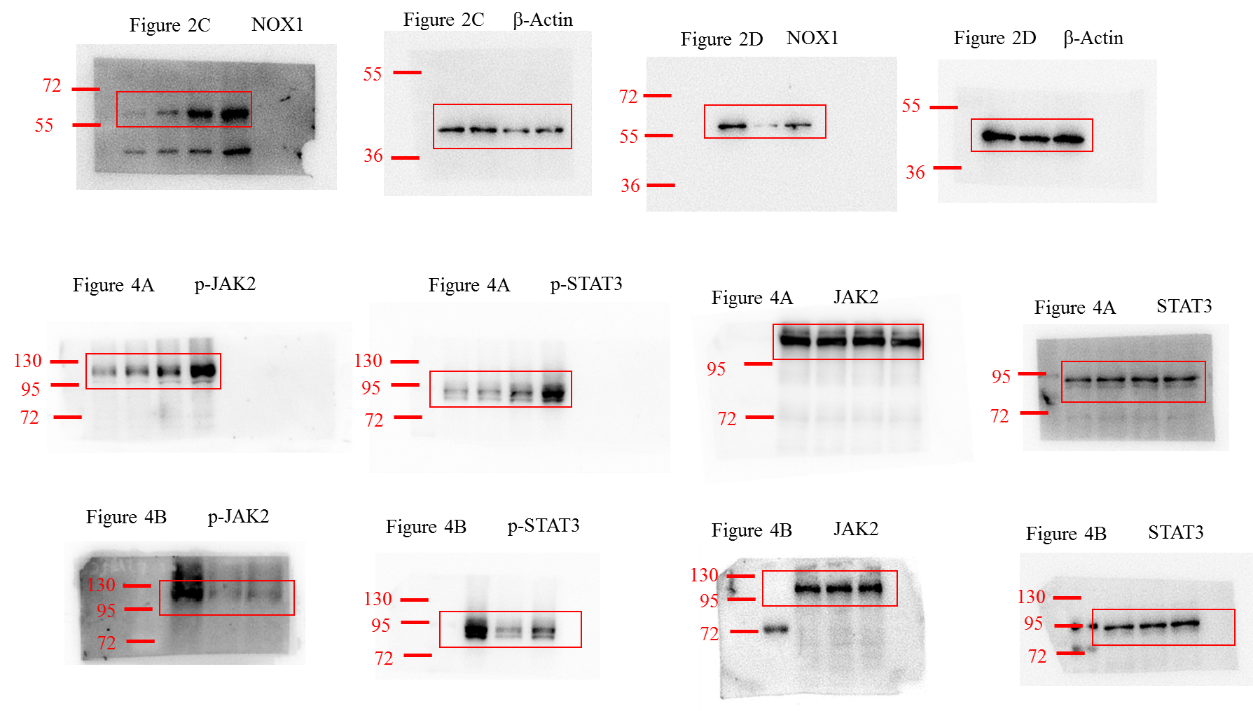


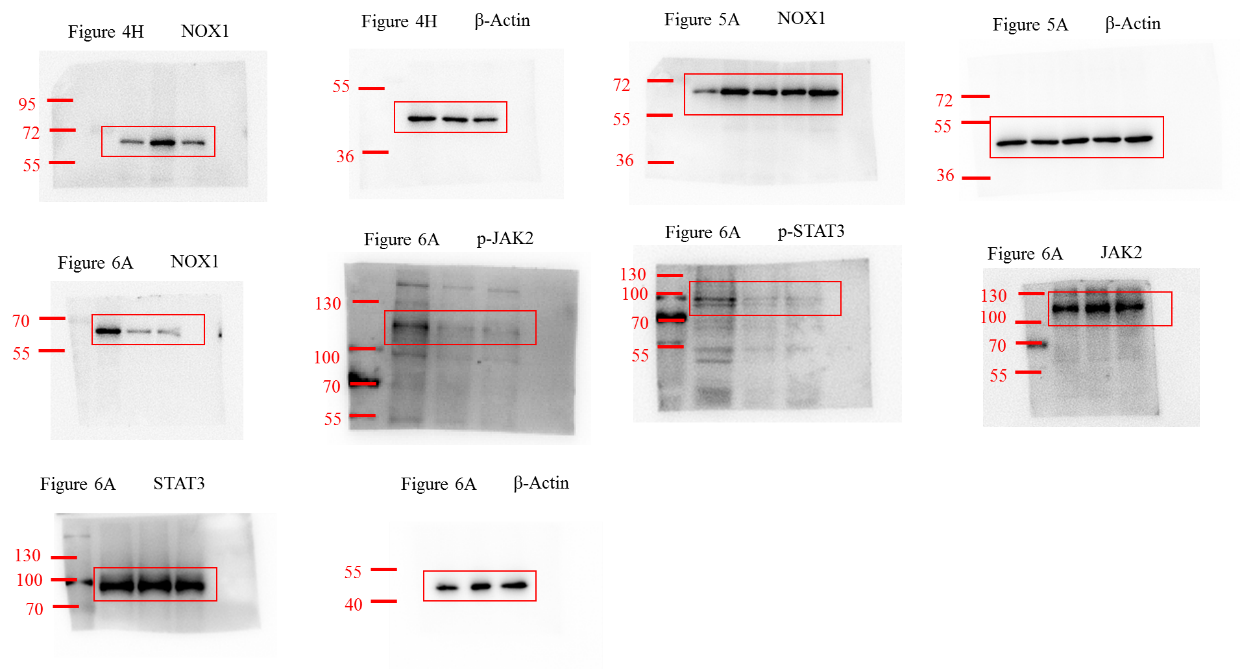


Supplementary Figure 7 Uncropped images of blots and gels in the article


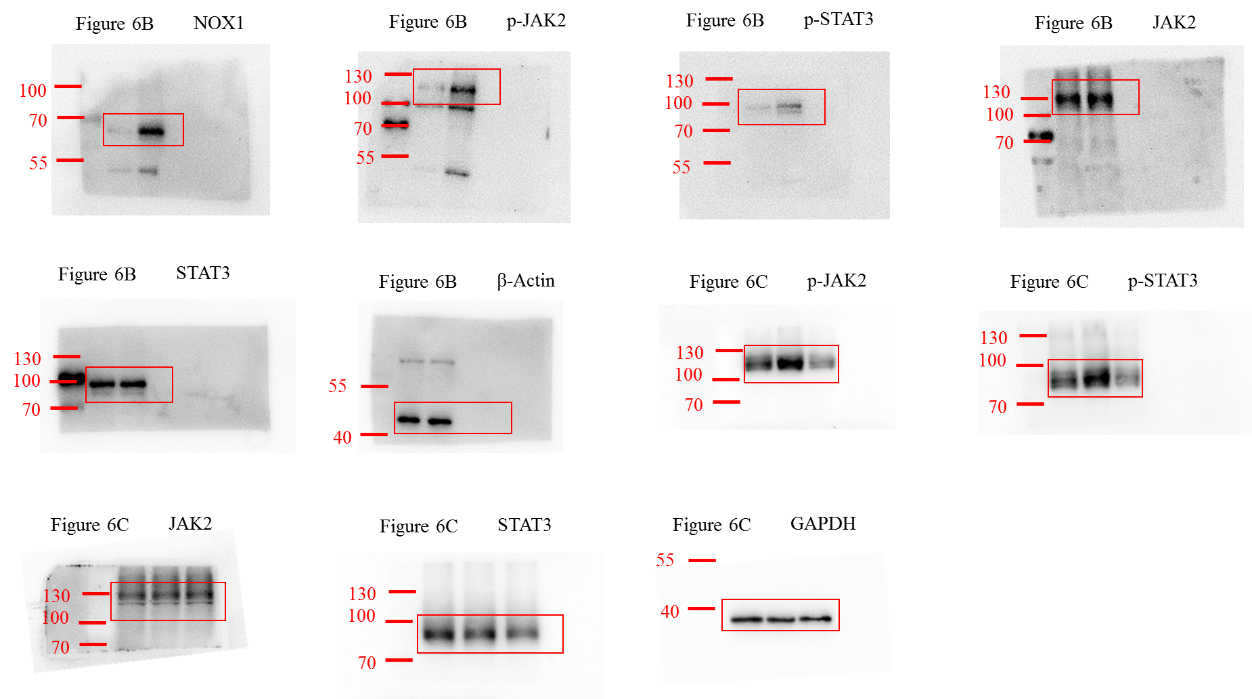


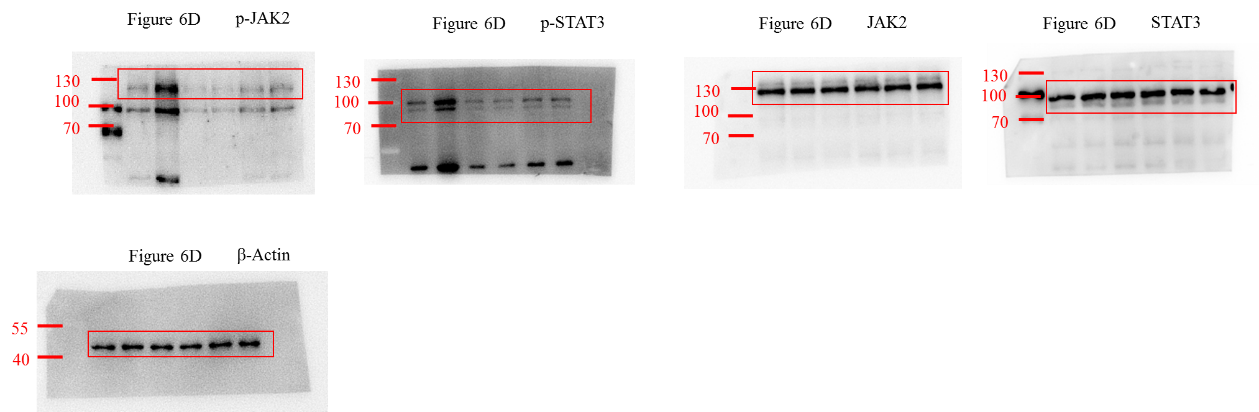


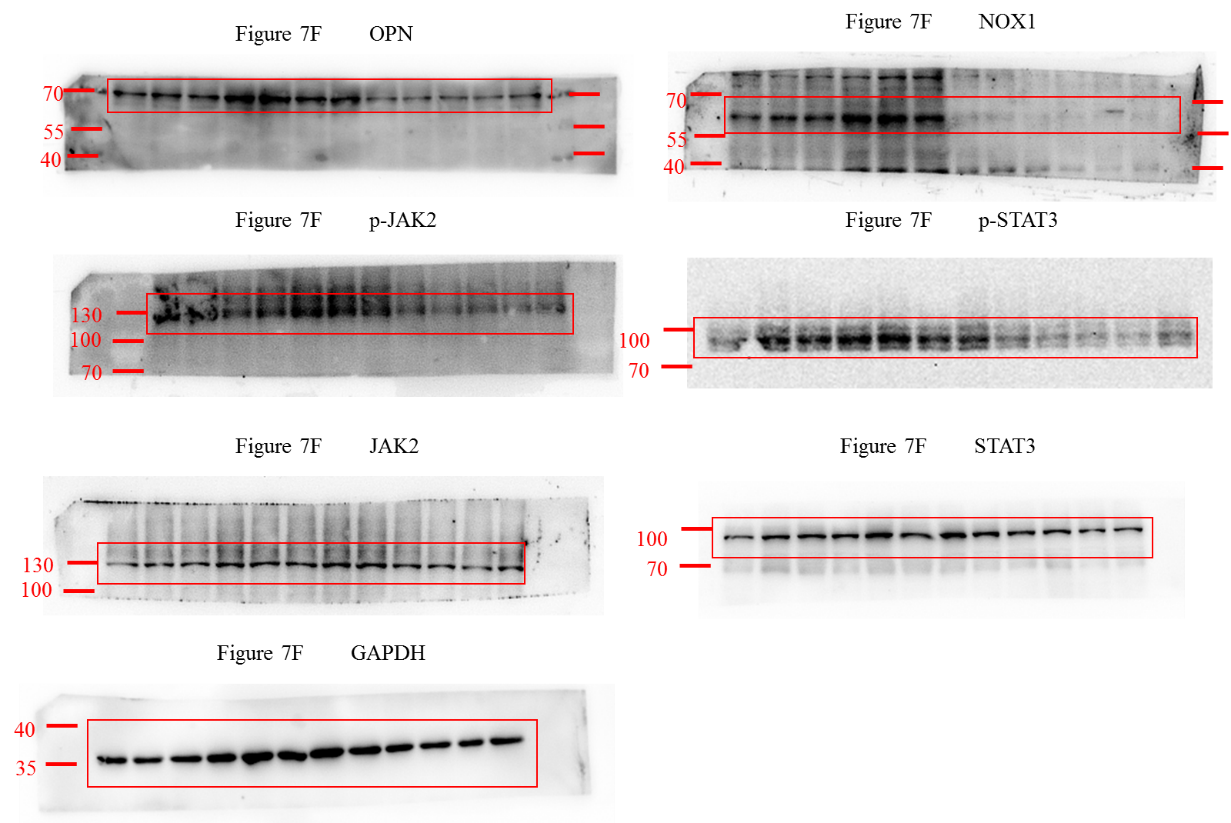


Supplementary Figure 7 Continued


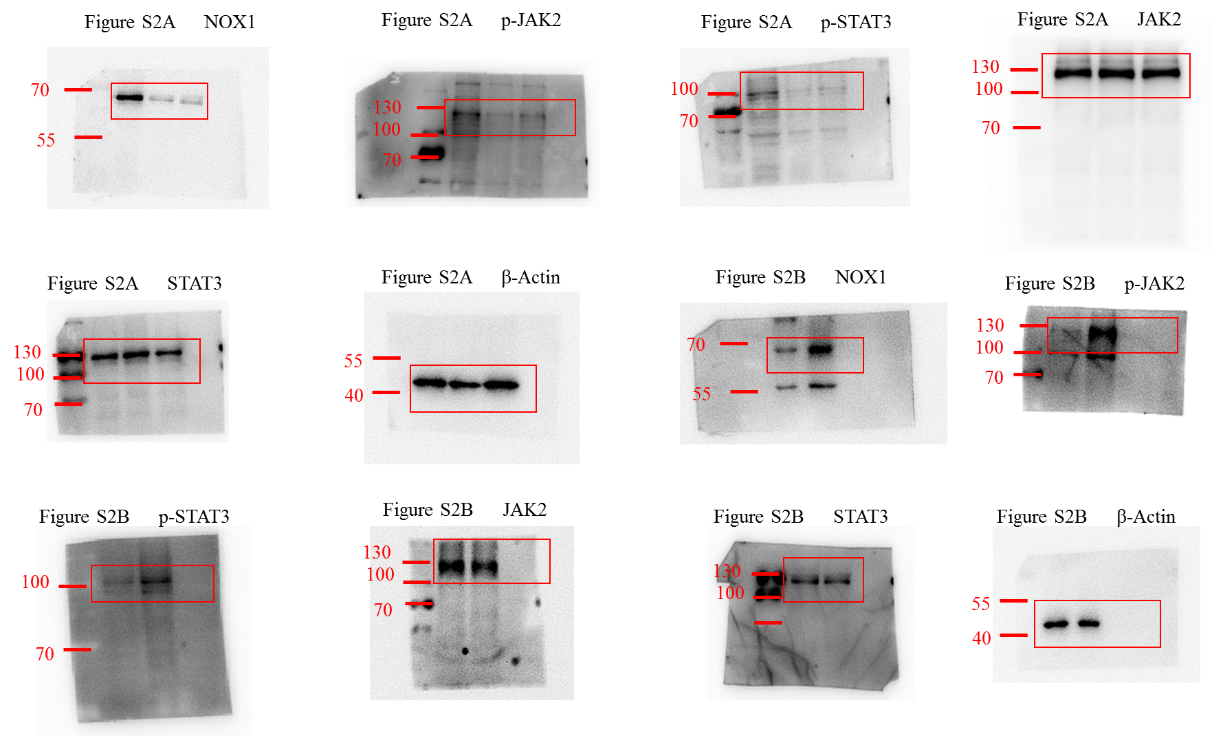


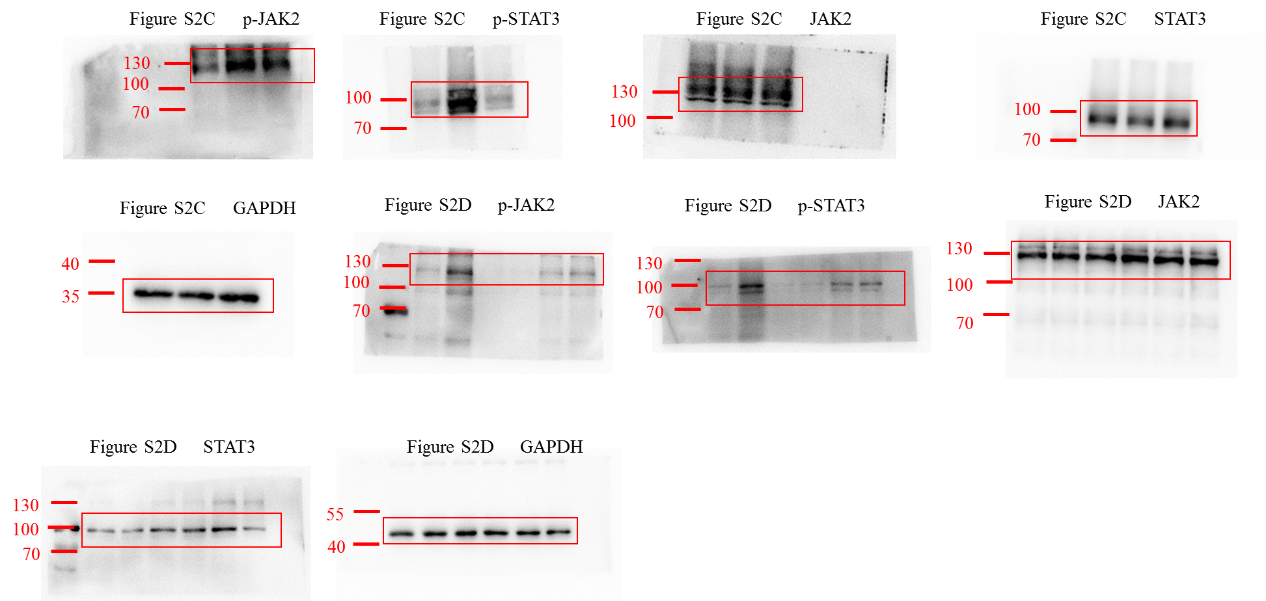


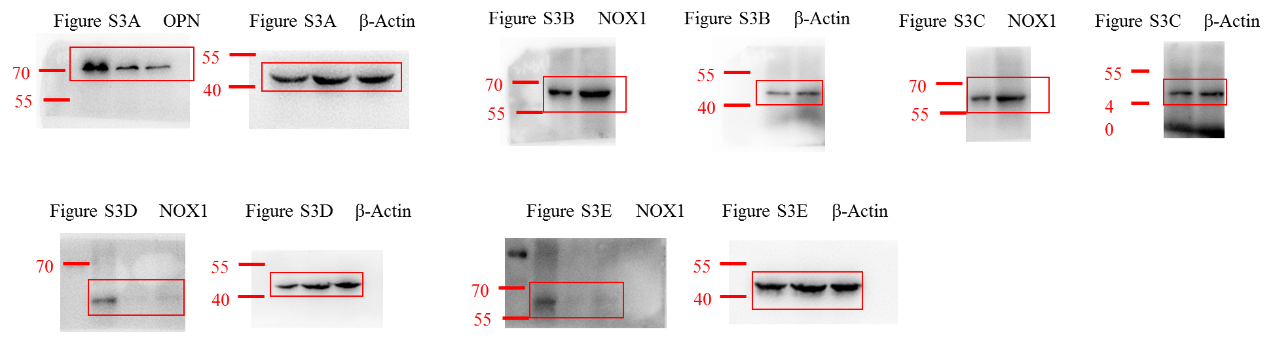


Supplementary Figure 7 Continued
